# Assessment of potential anti-cancer stem cell activity of marine algal compounds using an *in vitro* mammosphere assay

**DOI:** 10.1186/1475-2867-13-39

**Published:** 2013-05-01

**Authors:** Jo-Anne de la Mare, Jason N Sterrenberg, Mugdha G Sukhthankar, Maynard T Chiwakata, Denzil R Beukes, Gregory L Blatch, Adrienne L Edkins

**Affiliations:** 1 The Biomedical Biotechnology Research Unit (BioBRU), Department of Biochemistry, Microbiology and Biotechnology, Rhodes University, P. O. Box 94, Grahamstown, 6140, South Africa; 2 Division of Pharmaceutical Chemistry, Faculty of Pharmacy, Rhodes University, Grahamstown, South Africa; 3 College of Health and Biomedicine, Victoria University, Melbourne, Australia

**Keywords:** Mammosphere assay, Cancer stem cells, Halogenated monoterpenes

## Abstract

**Background:**

The cancer stem cell (CSC) theory proposes that tumours arise from and are sustained by a subpopulation of cells with both cancer and stem cell properties. One of the key hallmarks of CSCs is the ability to grow anchorage-independently under serum-free culture conditions resulting in the formation of tumourspheres. It has further been reported that these cells are resistant to traditional chemotherapeutic agents.

**Methods:**

In this study, the tumoursphere assay was validated in MCF-7 cells and used to screen novel marine algal compounds for potential anti-cancer stem cell (CSC) activity *in vitro*.

**Results:**

MCF-7 breast cancer cells were observed to generate tumourspheres or mammospheres after 3-5 days growth in anchorage-independent conditions and an apparent enrichment in potential CSCs was observed by an increase in the proportion of CD44^high^/CD24^low^ marker-bearing cells and Oct4 expression compared to those in the bulk population grown in regular adherent conditions. Using this assay, a set of algal metabolites was screened for the ability to inhibit mammosphere development as a measure of potential anti-CSC activity. We report that the polyhalogenated monoterpene stereoisomers RU017 and RU018 isolated from the red alga *Plocamium cornutum*, both of which displayed no cytotoxicity against either adherent MCF-7 breast cancer or MCF-12A non-transformed breast epithelial cells, were able to prevent MCF-7 mammosphere formation *in vitro*. On the other hand, neither the brown algal carotenoid fucoxanthin nor the chemotherapeutic paclitaxel, both of which were toxic to adherent MCF-7 and MCF-12A cells, were able to inhibit mammosphere formation. In fact, pre-treatment with paclitaxel appeared to enhance mammosphere formation and development, a finding which is consistent with the reported resistance of CSCs to traditional chemotherapeutic agents.

**Conclusion:**

Due to the proposed clinical significance of CSC in terms of tumour initiation and metastasis, the identification of agents able to inhibit this subpopulation has clinical significance.

## Background

The cancer stem cell theory challenges the traditional monoclonal models of cancer development and has revolutionized the way we think about the origin and progression of cancers [1,2]. This theory states that tumours consist of a heterogenous population of cells that is initiated and maintained by a subpopulation of cells with both cancer and stem cell characteristics [2,3]. Most importantly the theory purports that cancer stem cells (CSCs) are able to undergo assymetrical division, meaning that they can both self-renew to produce more cancer stem cells and differentiate to give rise to the various cell types within a tumour [1,4].

The first solid tumour stem cells were identified in breast cancer, where it was demonstrated that a CD44^+^CD24^-^ marker-bearing subpopulation could regenerate a tumour from as little as 100 cells, whereas tens of thousands of cells from the bulk population failed to do so [5]. Breast cancer stem cells (BCSCs) have been isolated from both primary patient samples and cell lines such as MCF-7 and SKBR3 [5-8]. These CSCs can be isolated in a number of ways. Originally BCSCs were identified and extracted from the bulk population using fluorescence-activated cell sorting (FACS) based on their specific cell surface markers, in this case CD44^+^CD24^-^lin^-^[5,8]. Recently, breast cancer stem-like cells have also been isolated based on their functional characteristics; in particular their ability to grow anchorage-independently in serum-free conditions [7,9]. In these culture conditions non-stem cancer cells undergo anoikis, a programmed cell death associated with loss of adhesion, thus selecting for the CSC-like subpopulation [10,11]. These CSC-like cells form tumourspheres or mammospheres in suspension *in vitro*, have been shown to be capable of *in vivo* tumour formation at limiting cell dilutions and express high levels of stem cell markers such as Oct4 [7,9,12].

While attention in the past decades has turned towards marine natural products as a source of lead anti-cancer compounds, marine algae have received considerably less attention in terms of their potential for bioactive metabolites than other marine organisms such as sponges, Cnidarians and cyanobacteria [13]. In addition, very few studies of the biological activity of algal metabolites go beyond the standard *in vitro* cytotoxicity screening tests [14,15]. Recently, a number of polyhalogenated monoterpene compounds were isolated from the red algae *Plocamium suhrii, Plocamium cornutum* and *Plocamium corallorhiza* collected from the South African coastline, which were cytotoxic to oesophageal and breast cancer cells *in vitro*[16,17]. We report that two polyhalogenated monoterpenes isolated from *Plocamium cornutum* red algae inhibit MCF-7 mammosphere formation *in vitro*, while having no adverse effects on either the bulk MCF-7 population or non-transformed MCF-12A breast epithelial cells.

## Results

### Paclitaxel and fucoxanthin, but not the monoterpenes RU017 and RU018, are toxic towards breast cancer and non-transformed breast epithelial cells *in vitro*

The differential toxicity of the algal polyhalogenated monoterpenes RU017 and RU018, as well as the carotenoid fucoxanthin (FXN) and the chemotherapeutic paclitaxel (Ptx), against breast cancer and non-transformed breast epithelial cells was assessed by MTT assay. In this assay, Ptx was found to decrease the percentage survival of immortalized non-transformed breast epithelial cells (MCF-12A) to approximately 70% at a concentration equivalent to the IC_50_ value of the compound in both metastatic (MDA-MB-231) and non-metastatic (MCF-7) breast cancer cells *in vitro* (Table 1). In the case of FXN, the metastatic MDA-MB-231 cells were more susceptible to the carotenoid than non-metastatic MCF-7 cells, while MCF-12A breast epithelial cells displayed a moderate susceptibility to the compound at a concentration of 10 μM. For the halogenated monoterpenes RU017 and RU018, neither of the compounds was toxic to MCF-7 or MDA-MB-231 breast cancer or MCF-12A non-transformed breast epithelial cell lines, even at a concentration of 300 μM (Table 1).

**Table 1:** **Differential cytotoxicity screening of paclitaxel and novel algal compounds against breast cancer and non-transformed breast epithelial cells *in vitro***

					
**Compound**	**Structure**	**MDA-MB-231**	**MCF-7**	**MCF-12A**	
		**IC**_**50 **_**value**		**Concentration**	**Percentage survival**
Paclitaxel		112.82 ± 5.22 nM	94.97 ± 1.10 nM	100 nM	69.40 ± 2.8%
Fucoxanthin		11.07 ± 0.56 μM	23.00 ± 0.01 μM	10 μM	71.32 ± 9.2%
RU017		Non-toxic	Non-toxic	300 μM	98.11 ± 4.6%
RU018		Non-toxic	Non-toxic	300 μM	95.15 ± 3.4%

### MCF-7 breast cancer cells form mammospheres in anchorage-independent serum-free culture conditions

**Figure 1:**
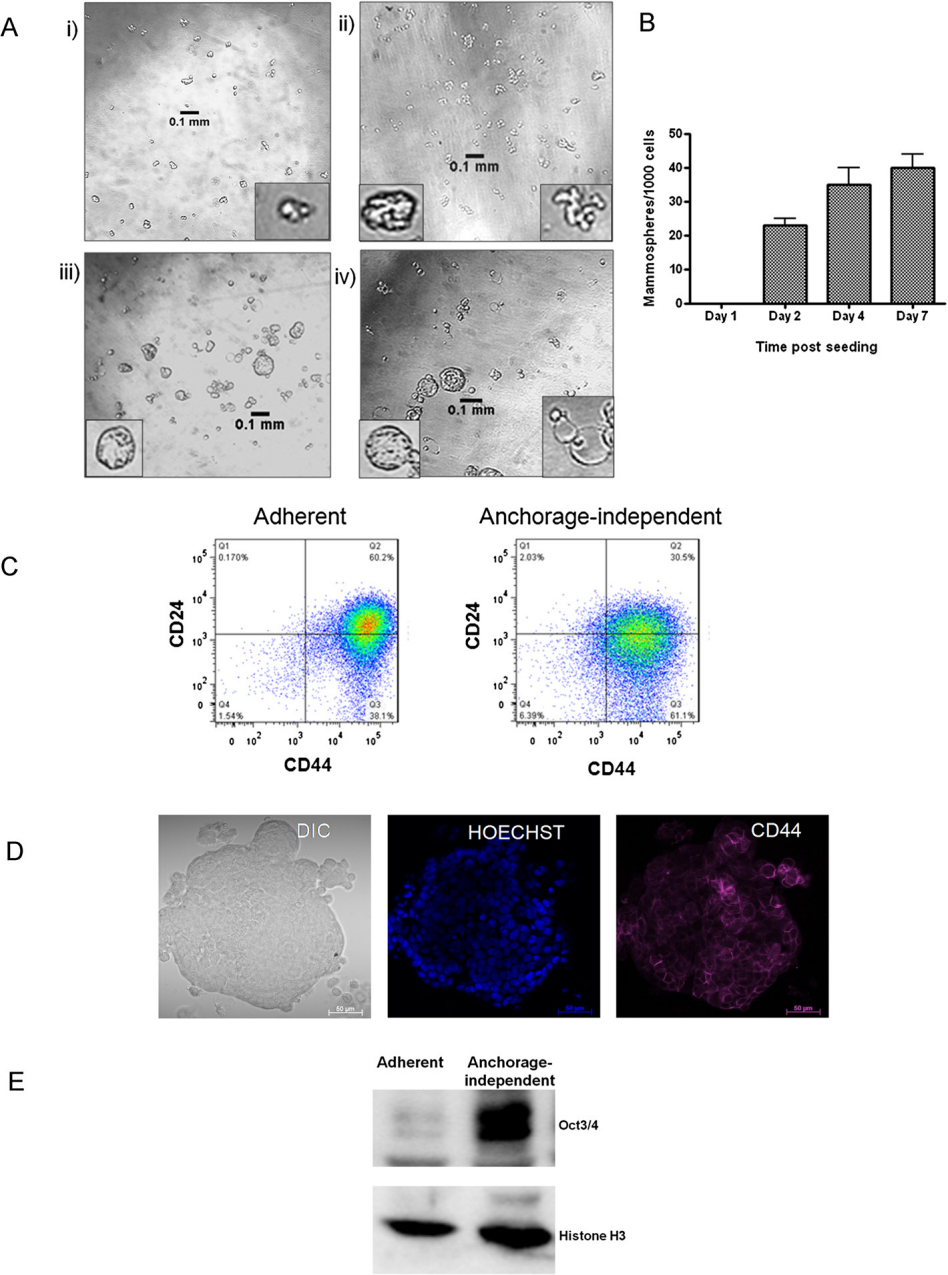
**breast cancer cells form cancer stem cell-enriched mammospheres under serum-free culture conditions. A**) Alterations in number, size and shape of mammospheres was observed over the course of seven days. Photographs were taken at Day 1 (i), Day 3 (ii), Day5 (iii) and Day 7 (iv). Images are representative of at least three randomly selected fields. Insets show specific structural features of MCF-7 mammospheres at various stages of their development. Images were captured under a light microscope at 100× magnification using a Nikon Coolpix 990 camera. Scale bars representative of 100 *μ*m. Note: Insets not set to scale. **B**) Sphere forming efficiency SFE was calculated as the number of mammospheres/spheres (average diameter = 100 μm) formed in 96 wells plated with a single cell divided by the original number of single cells seeded and expressed as a percentage. Error bars represent the standard error of the mean where n = 3. **C**) Flow cytometry histogram showing an increase in the CD44^high^/CD24^low^ cell surface marker-bearing subpopulation between adherent and mammosphere-derived (anchorage-independent) cells generated using FlowJo software (Tree Star Inc.). **D**) Confocal microscopic analysis of anchorage-independent cells where Hoechst 33342 was used to stain nuclei (blue) while CD44 was stained with an allophycocyanin-conjugated antibody (purple). Images were captured using a Zeiss LSM510 Meta confocal microscope. Scale bars represent 20 *μ*m. **E**) Western analysis to assess Oct3/4 expression in mammospheres-derived cells compared to the bulk MCF-7 population. Western analyses were carried out on a total of 30 μg of protein derived from whole cell lysates after cultivation in either regular adherent or serum-free anchorage-independent growth conditions. In each case, lysates were probed for histone H3 as a loading control.

The mammosphere assay was carried out for the MCF-7 breast cancer cell line in order to enrich for cancer stem cell-like cells, which are able to grow in anchorage-independent serum-free conditions [11]. The development of non-adherent tumourspheres or mammospheres [11] under these conditions was observed over the course of one week. As depicted in Figure 1, the single-cell suspension seeded on Day 0 had formed small, irregular cell “clumps” by Day 1 (Figure 1Ai), which had developed into small suspended colonies representative of mammospheres by Day 3 (Figure 1Aii). By Day 5, the mammospheres had increased in size to approximately 100 μM (0.1 mm) in diameter and displayed a more regular spherical three-dimensional shape (Figure 1Aiii). After seven days growth in anchorage-independent conditions, the mammospheres, while remaining roughly the same size as Day 5, began to exhibit different morphologies (Figure 1Aiv). The most striking feature of the Day 7 cultures was the formation of hollow mammospheres; empty bubble-like structures surrounded by one or more cells or small mammospheres attached to what appeared to be an outer membrane of the hollow spherical bodies (Figure 1Aiv, inset bottom right). The number of mammospheres per well increased steadily from Day 1 to Day 7 (Figure 1B), while the number of single cells and clusters containing one to three cells were observed to decrease with increased incubation time (*data not shown*).

The enrichment of putative CSCs in mammospheres assay was assessed by flow cytometry to detect specific CSC markers, revealing that mammosphere-derived cells (anchorage-independent) were enriched for CD44^high^/CD24^low^ CSC-like cells, displaying a 60.2% proportion of CD44^high^/CD24^low^ fluorescent events compared to 30.5% in the adherent bulk MCF-7 population (Figure 1C). The expression of CD44 cell surface marker in mammosphere-derived cells was confirmed by confocal microscopy using fluorescently tagged anti-CD44 antibodies (Figure 1D). Mammosphere-derived cells (anchorage-independent) also displayed increased expression of the stem cell marker Oct3/4 compared to those derived from the bulk adherent population, as assessed by Western analysis (Figure 1E).

### The polyhalogenated monoterpene stereoisomers RU017 and RU018 inhibit the formation of MCF-7 mammospheres

**Figure 2:**
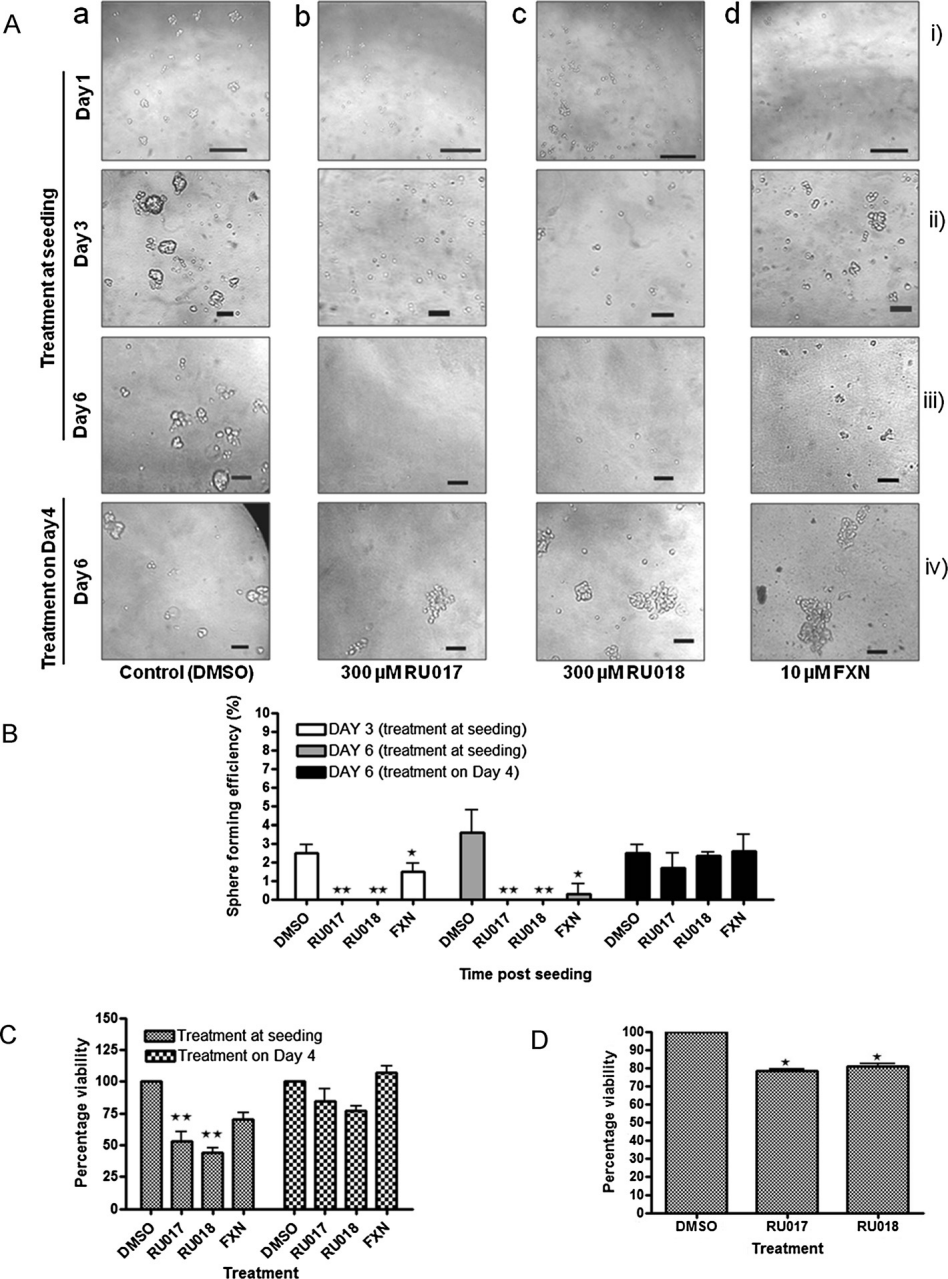
**The polyhalogenated monoterpenes RU017 and RU018 inhibit the formation but not development of MCF-7 mammospheres**. **A**) Mammosphere cultures were treated with either the DMSO vehicle control (**a**), 300 μM of either of the halogenated monoterpene stereoisomers RU017 (**b**) or RU018 (**c**) or10 μM fucoxanthin (FXN) (**d**). The latter concentrations were derived from the IC_50_ values in MDA-MB-231 or MCF7 breast cancer and MCF12A non-cancerous cells grown in anchorage-dependent conditions in the case of FXN. Images were captured under a light microscope at either 400× or 100× magnification using a Nikon Coolpix 990 camera on Day 1 (i), Day 3 (ii) and Day 6 (ii) after treatment with the relevant compounds upon seeding and on Day 6 for mammospheres treated with compounds four days after seeding (iv). Images were representative of at least three randomly selected fields for each treatment. Analysis of images and calculation of scale was carried out using ImageJ (NIH freeware). Scale bars representative of 0.1 mm. **B**) Sphere forming efficiency (SFE) and **C**) mammosphere viability on Day 8 as determined by WST-1 assay. **D**) Untreated mammospheres were dissociated and seeded as a single cell suspension into regular culture media containing either the vehicle control or 300 μM of RU017 or RU018, cultured under adherent conditions and assessed for viability by means of an MTT assay. The percentage viability in **C**) and **D**) was calculated relative to the DMSO vehicle-treated control (taken as 100%) when an equal numbers of cells are seeded and treated for the same period. For **B**), **C**) and **D**) error bars indicate the standard error of the mean where n = 3 (**B**) or 5 (**C** and **D**). Statistical significance was calculated using a Students *t*-test (**p < 0.01, *p < 0.05).

The effect of the marine algal polyhalogenated monoterpenes RU017 and RU018, as well as the carotenoid FXN, on the formation and development of MCF-7 mammospheres was assessed by addition of these compounds to the culture medium either at seeding or after four days growth in anchorage-independent conditions (Figure 2). The compounds were added at a concentration equivalent to the IC_50_ values in MDA-MB-231 cells as determined by MTT assay (Table 1). In the case of the non-toxic compounds RU017 and RU018, a concentration of 300 μM was selected. This high concentration of the compounds was not, however, toxic to the MCF-7 breast cancer or MCF-12A breast epithelial cell lines under regular adherent conditions (Table 1). However, under non-adherent conditions, both of the latter compounds prevented the formation of MCF-7 mammospheres, leaving only single cells or clusters of two or three cells in the treated samples by Day 3, which were further decreased by Day 6 (Figures 2Ab and 2Ac). In comparison, MCF-7 cells treated with the vehicle control (dimethyl sulphoxide, DMSO) formed distinct mammospheres by Day 3 (Figure 2Aa ii). The carotenoid FXN caused a statistically significant decrease in the sphere forming efficiency (SFE) both at Day 3 and Day 6 (Figure 2B) and resulted in smaller mammospheres in general compared to the control (Figure 2Ad vs. Figure 2Aa). The compound was, however, unable to inhibit mammosphere formation completely (Figures 2Ad and 2B).

For all of the algal compounds tested, the effect of the compounds on sphere forming efficiency when added at Day 4 differed from that obtained when the compounds were added upon seeding of the MCF-7 cells (Figure 2A iii vs. iv). When added to existing MCF-7 mammospheres on Day 4, none of the compounds screened were able to remove the existing MCF-7 mammospheres or prevent their further development (Figures 2A iv and 2B). The addition of FXN after four days resulted in the formation of much larger, irregularly shaped mammospheres than those observed in the DMSO-treated MCF-7 cells (Figure 2Ad iv vs. Figure 2Aa iv). In contrast to the effects when added upon seeding, neither of the stereoisomers RU017 or RU018 were able to eliminate the MCF-7 mammospheres or affect their further development when added at Day 4 (Figures 2Ab iv and 2Ac iv, respectively).

The WST-1 cell proliferation assay was carried out on treated mammospheres (treated both at seeding and on Day 4) after eight days growth in anchorage-independent culture conditions. The percentage survival values for each sample were calculated relative to the DMSO-treated vehicle control (taken as 100%) after 8 days growth in anchorage-independent conditions when equal numbers of cells are seeded, and are indicated in Figure 2C. While FXN produced a (non-significant) reduction in cellular survival to 70% when added upon seeding, the compound was unable to decrease cell survival when added four days after seeding (107% survival relative to DMSO-treated cells). The halogenated monoterpenes RU017 and RU018 both resulted in statistically significant decreases in cellular survival (53% and 44%, respectively) when added at Day 0, while the slight reduction in survival (to 84% and 77%, respectively), when added at Day 4 was not statistically significant.

In order to further characterize the effect of RU017 and RU018 on sphere forming efficiency as observed in Figure 2B, untreated mammospheres were dissociated and seeded into regular anchorage-dependent growth conditions, followed by treatment with either of the algal compounds and assessed for viability using an MTT assay. In this assay, it was found that the algal compounds had a small but statistically significant effect on cell viability when compared to the vehicle control, but that neither were able to reduce cell viability to below 78% (Figure 2D). This minor reduction in viability was similar to that observed when cells were treated with the monoterpenes in anchorage-independent mammosphere conditions after four days growth, but differed from that observed when cells were treated upon seeding into anchorage-independent culture conditions (Figure 2C).

### The inhibitory effect of the marine algal compounds RU017 and RU018 on MCF-7 mammosphere formation *in vitro* is dose-dependent

**Figure 3:**
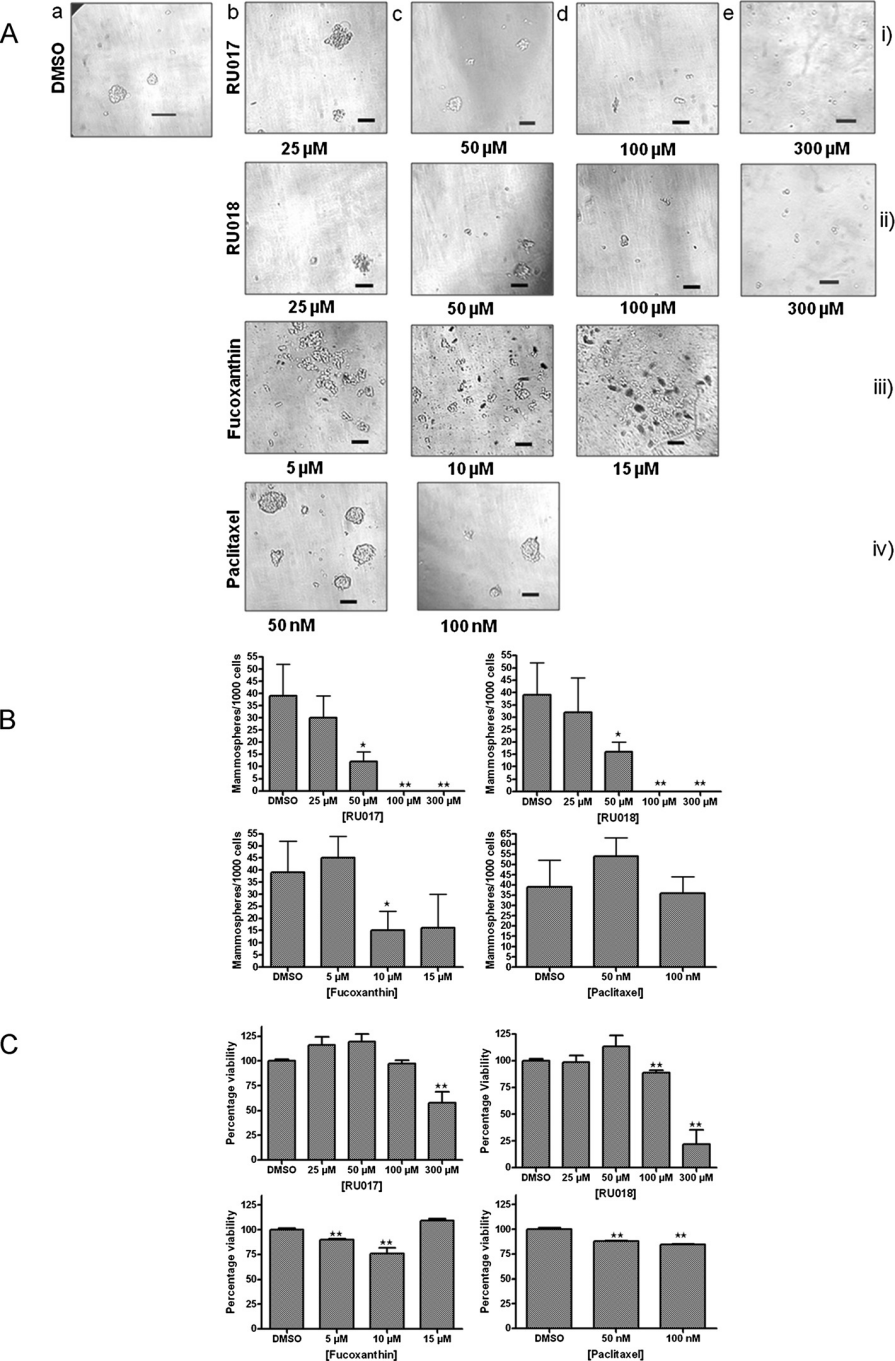
**The inhibitory effect of RU017 and RU018 on MCF-7 mammosphere formation is dose-dependent. A**) Photographs of mammospheres formed after six days incubation in anchorage-independent serum-free conditions. MCF-7 cells were seeded as a single cell suspension into media containing either (a) DMSO vehicle control or a range of concentrations of the halogenated monoterpene stereoisomers (i) RU017 or (ii) RU018 [(b)25, (c)50, (d)100 or (e)300 μM], (iii) fucoxanthin [(b)5, (c)10 or (d)15 μM] or (iv) paclitaxel [(b)50 or (c)100 nM]. The latter concentrations of compounds tested was informed by the cytotoxicity values observed under adherent conditions. Cells were photographed under a light microscope at 100× magnification. All images are representative of at least three randomly selected fields and were set to the same scale using ImageJ (NIH freeware). Scale bars representative of 0.1 mm. **B**) Quantification of the mammospheres generated in terms of sphere forming efficiency (SFE) after six days for the various treatments. SFE was calculated as the number of spheres (average diameter = 100 μm) formed in 96 wells plated with a single cell divided by the original number of single cells seeded and expressed as a percentage. **C**) Cell viability in treated mammosphere samples as assessed by WST-1 assay. The percentage viability after each of the treatments in **C**) was calculated relative to the DMSO-treated negative control (taken as 100%) after 8 days growth when an equal numbers of cells are seeded. Error bars indicate standard deviation where n = 3 (**B**) or 5 (**C**). Statistical significance of the differences in SFE and percentage survival relative to the DMSO control were calculated using a Students *t*-test (**p < 0.01, *p < 0.05).

The effects of the algal compounds RU017, RU018 and FXN on the formation and development of MCF-7 mammospheres were more thoroughly investigated by determining whether the observed alterations to the mammospheres were dose-dependent. In addition, the effect of various concentrations of the chemotherapeutic agent, Ptx, on sphere forming efficiency was assessed. For both of the monoterpene stereoisomers, RU017 and RU018, the inhibition of MCF-7 mammosphere formation appeared to be dose-dependent (Figures 3A i and ii, respectively; Figure 3B). In each case, treatment with 50 μM, but not 25 μM, of the compounds had a significant effect on the number (Figure 3B) and size of the MCF-7 mammospheres formed after six days, although the mammospheres treated with 25 μM were observed to be more irregular in shape when compared to the DMSO-treated control (Figures 3Ab and 3Ac, i and ii respectively). The latter concentrations of RU017 and RU018 did not, however, reduce cellular viability of the treated mammospheres compared to the DMSO control as determined by WST-1 assay (Figure 3C). For both halogenated monoterpenes, treatment with 100 μM appeared to inhibit mammosphere formation, resulting only in small cell clumps (Figure 3Ad, i and ii, respectively). However, in the WST-1 assay, the decrease in percentage viability relative to the control was statistically significant only in the case of RUMB-018 (Figure 3C). Treatment of MCF-7 cells upon seeding in anchorage-independent conditions with 300 μM of either RU017 or RU018 prevented mammosphere formation entirely and significantly reduced cell viability for both compounds (Figures 3Ae i and ii, respectively; Figures 3B and C).

In the case of the carotenoid compound FXN, none of the concentrations tested were able to completely eliminate mammosphere formation when added to MCF-7 cells upon seeding into anchorage-independent conditions, although a dose-dependent decrease in mammosphere size was observed (Figure 3A iii). The effects of FXN on sphere forming efficiency and cell viability, however, were not dose-dependent (Figures 3B and C). For all concentrations tested, FXN was unable to reduce cell viability to below 76% relative to the DMSO-treated control (Figure 3C).

The chemotherapeutic drug Ptx appeared to increase the number of MCF-7 mammospheres when 50 nM was added upon seeding (Figures 3Ab iv and 3B), while treatment with 100 nM had little effect on sphere forming efficiency compared to the DMSO-treated control (Figures 3Ac iv and 3B). This was despite the latter concentration being reported as the IC_50_ value for MCF-7 cells under adherent conditions [18]. In comparison, using the WST-1 cytotoxicity assay, Ptx led to a minor but statistically significant reduction in mammosphere viability when added at both 50 and 100 nM in anchorage-independent mammosphere conditions. Importantly, neither of the concentrations tested were able to reduce cellular viability to below 84% (Figure 3C).

### Pre-treatment of adherent MCF-7 cultures with algal compounds or Ptx does not prevent mammosphere formation

The compounds RU017, RU018, FXN and Ptx were used to pre-treat MCF-7 cells grown under normal anchorage-dependent conditions prior to seeding under anchorage-independent mammosphere conditions. The consequences of such treatment on mammosphere development and viability were compared to those obtained for treatment of mammospheres at seeding (Day 0) or after four days growth in mammosphere conditions (Day 4).

**Figure 4:**
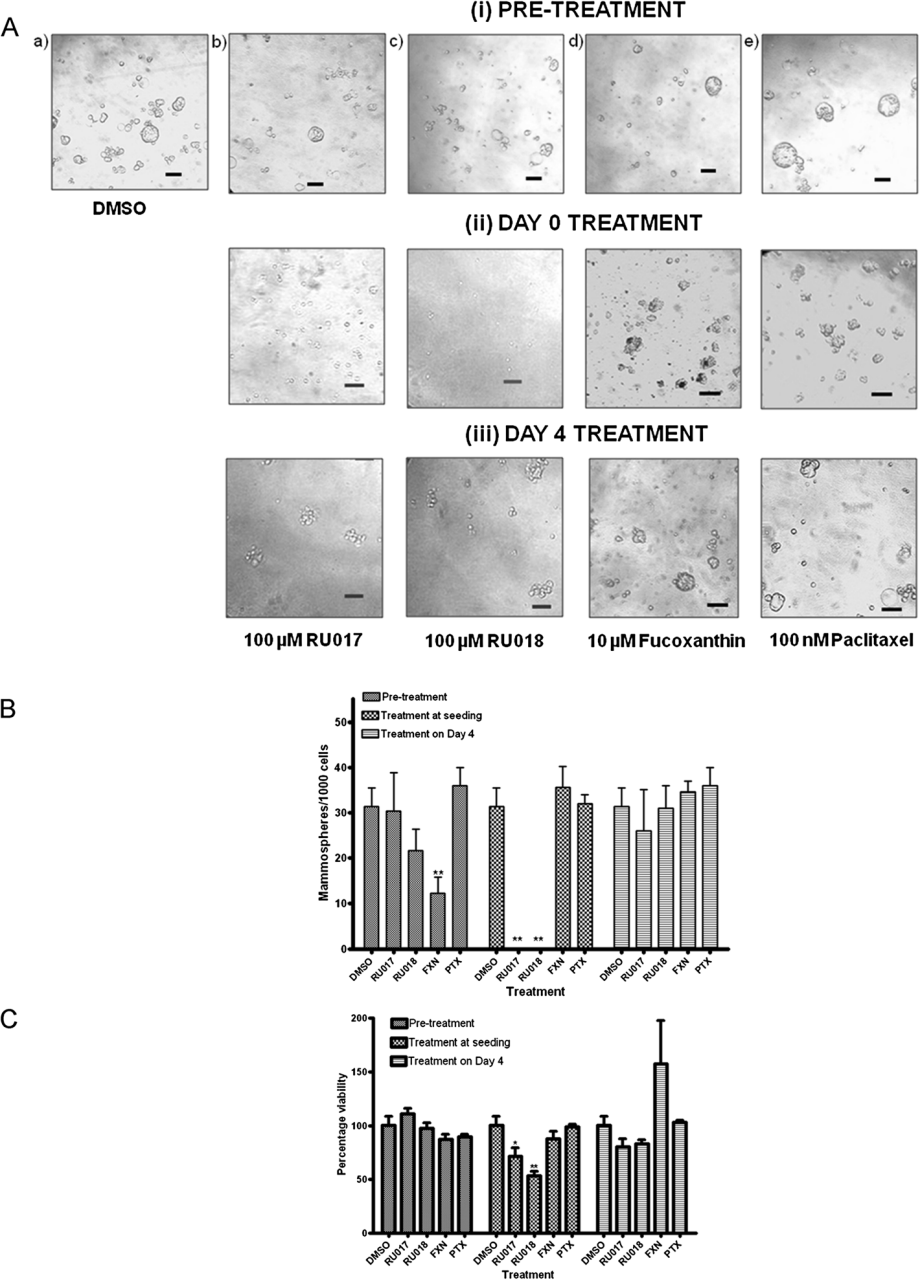
**Pre-treatment of MCF-7 cells with RU017/RU018 under adherent conditions is unable to prevent mammosphere formation. A**) Treatment of MCF-7 cells was with a) DMSO vehicle control or either of the halogenated monoterpene stereoisomers b) RU017 or c) RU018 (100 μM), d) fucoxanthin (10 μM) or e) paclitaxel (100 nM). For pre-treatment, MCF-7 cells were cultured in regular anchorage-dependent culture conditions containing the compound of interest, before trypsinizing and transferring to anchorage-independent serum-free mammosphere conditions as a single cell suspension. The Day 0 and Day 4 samples refer to treatment of MCF-7 cells either upon seeding or after 4 days growth, respectively, in anchorage-independent serum-free culture conditions. Images were captured under a light microscope at 100× magnification. Each image is representative of at least 3 randomly selected fields. Images were set to the same scale using ImageJ (NIH freeware). Scale bars are equivalent to 0.1 mm. **B**) Quantification of mammospheres formed in terms of sphere forming efficiency (SFE) after six days for each treatment. SFE was calculated as the number of spheres (average diameter = 100 μm) formed in 96 wells plated with a single cell divided by the original number of single cells seeded and expressed as a percentage. **C**) Assessment of cell viability in treated mammospheres by WST-1 assay. The percentage viability after each of the treatments in **C**) was calculated relative to the DMSO-treated negative control (taken as 100%) after 8 days growth when an equal numbers of cells are seeded. In both **B**) and **C**), error bars indicate the standard error of the mean where n = 3 (B) or 5 (**C**). Statistical significance of the differences in SFE and percentage survival relative to the DMSO control were calculated using a Students *t*-test (**p < 0.01, *p < 0.05).

For all four of the compounds RU017, RU018, FXN and Ptx, pre-treatment of MCF-7 cells in anchorage-dependent culture conditions prior to seeding into anchorage-independent conditions in the mammosphere assay was unable to either prevent mammosphere formation (Figure 4A i) or effectively reduce cell viability relative to the DMSO control (Figure 4C). Although pre-treatment with FXN resulted in a statistically significant decrease in sphere forming efficiency after 6 days incubation in anchorage-independent conditions (Figure 4B), the compound did not completely inhibit mammosphere formation (Figure 4Ai d) or reduce cellular viability of pre-treated mammospheres (Figure 4C). Interestingly, pre-treatment with the chemotherapeutic agent Ptx, though having little effect on sphere forming efficiency (Figure 4B), appeared to produce larger, more regular shaped mammospheres when compared to the DMSO vehicle-treated control (Figure 4Ai e vs. a), while having little effect on cell viability (Figure 4C).

### Treatment of second and third generation MCF-7 mammospheres with the algal compounds RU017 and RU018 causes a dose-dependent decrease in sphere forming efficiency

**Figure 5:**
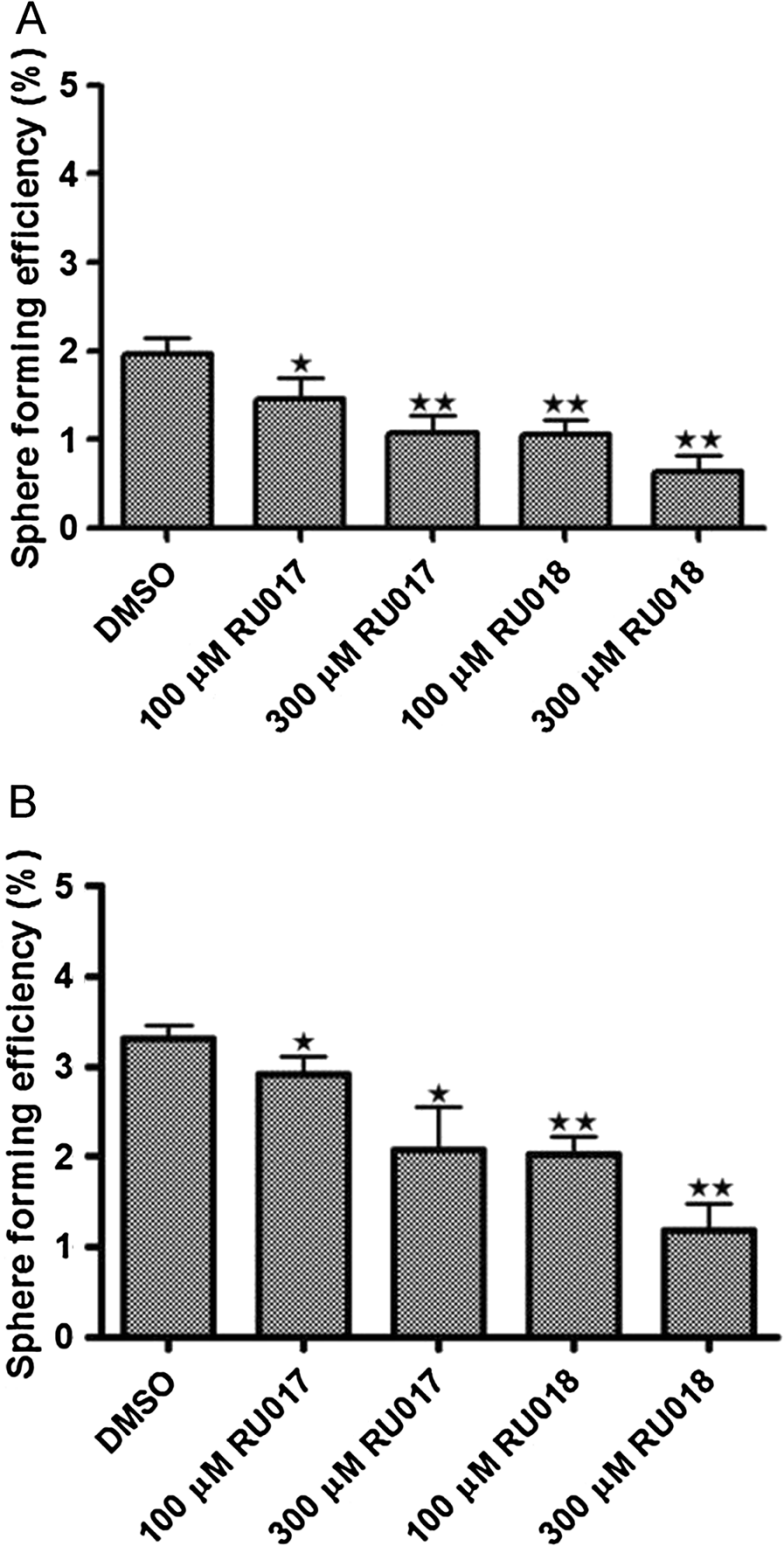
**The halogenated monoterpenes RU017 and RU018 have a dose-dependent inhibitory effect on second and third generation mammosphere formation.** Untreated mammospheres of first and second generation were dissociated and reseeded into anchorage-independent conditions in serum-free culture media containing the compounds of interest in order to assess second (**A**) and third generation (**B**) sphere forming efficiency (SFE), respectively. In both cases, mammosphere-derived MCF-7 cells were seeded as a single cell suspension into media containing either the DMSO vehicle control or either 100 or 300 μM of the halogenated monoterpene stereoisomers RU017 or RU018. SFE was calculated as the number of spheres (average diameter = 100 μm) formed after six days in 96 wells plated with a single cell divided by the original number of single cells seeded and expressed as a percentage. Error bars indicate the standard error of the mean where n = 5. Statistical significance of the differences in SFE relative to the DMSO control were calculated using a Students *t*-test (**p < 0.01, *p < 0.05).

The inhibitory effect on MCF-7 mammosphere formation by the halogenated monoterpenes RU017 and RU018 was further assessed by screening these compounds against second and third generation mammospheres. Untreated mammospheres were dissociated, reseeded under anchorage-independent conditions and treated with either 100 μM or 300 μM of RU017 or RU018. In both cases, although the compounds were unable to completely prevent MCF-7 mammosphere formation as was the case for primary mammospheres, treatment upon seeding of second and third generation mammospheres resulted in a dose-dependent decrease in sphere forming efficiency (Figure 5A and B). However, the decrease in SFE was greater for both compounds in the second vs. the third generation mammospheres. The inhibitory effect on mammosphere formation was not equal between the stereoisomers, with RU018 being more effective than RU017 in inhibiting both second and third generation mammosphere formation (Figure 5A and B). However, neither of the compounds had a significant effect on cell viability in either second or third generation mammosphere assays (data not shown).

## Discussion

In this study, the tumoursphere or mammosphere system was used to allow for the screening of marine algal compounds for potential anti-cancer stem cell (CSC) activity. The relatively small number of mammospheres formed compared to the total of 1000 cells seeded, as well as the enrichment in Oct4 expression and CD44^high^/CD24^low^ marker-bearing cells in isolated mammospheres, lent credence to the hypothesis that only a specific subset of the bulk MCF-7 population, displaying unique functional and phenotypic characteristics, was capable of propagation in suspension and validated the mammosphere assay employed [7,19]. While the addition of the test compounds at the stage of seeding of MCF-7 cells into anchorage-independent culture conditions assessed the effect on mammosphere formation, dosing with the compounds to existing mammospheres on Day 4 measured the ability to remove the mammospheres or alter further development. In addition, pre-treatment with compounds prior to seeding into mammosphere conditions provided preliminary insight as to the mechanism of action, in particular the ability to remove CSCs from the bulk population when grown under adherent conditions.

The commonly used chemotherapeutic agent, Ptx, was unable to prevent mammosphere formation when added at a concentration which was toxic to both breast cancer and non-transformed breast epithelial cells under adherent conditions, nor was the drug able to reduce mammosphere viability to below 84%. This is consistent with previous reports which demonstrated that 10 ng/L (12 pM) of Ptx had no effect on MDA-MB-231 mammosphere survival [20]. In addition, when added at a concentration of roughly half the IC_50_ value of MCF-7 cells, Ptx appeared to promote mammosphere formation and pre-treatment of MCF-7 cells prior to seeding produced larger, more defined mammospheres. This data suggests that traditional chemotherapy regimens such as Ptx may be ineffective against cancer cells growing in suspension (as occurs in the bloodstream during metastasis) or that the cancer stem cell sub-population may be resistant to these agents, as has been suggested in the literature [21]. However, significant further work is required in order to substantiate these hypotheses.

The carotenoid compound, fucoxanthin (FXN), was shown to be toxic to MDA-MB-231 and MCF-7 breast cancer cells and, to a lesser extent, non-cancerous MCF12A breast epithelial cells. However, the compound was unable to prevent the formation of MCF-7 mammospheres when added either upon seeding or at Day 4, or when used to pre-treat MCF-7 cells prior to seeding into anchorage-independent mammosphere conditions. The inability of FXN to inhibit mammosphere formation and development revealed that the anti-mammosphere activity was not a feature shared by all compounds of marine algal origin.

In the case of the halogenated monoterpene stereoisomers RU017 and RU018, both of which were shown to have no effect on the survival and proliferation of either breast cancer cells or non-transformed breast epithelial cells *in vitro*, the compounds were able to completely inhibit mammosphere formation when added to MCF-7 cells upon seeding in the mammosphere assay. Since mammosphere formation is reportedly dependent on the presence of CSC [9] and isolated mammospheres in this study showed an increase in cells expressing the CD44^+^/CD24^-^ breast cancer stem cell (BCSC) phenotype, the ability of RU017 and RU018 to inhibit mammosphere formation suggested that these compounds have putative anti-CSC activity and may inhibit a signal transduction pathway which is essential in mammosphere formation. Compounds RU017 and RU018 were not, however, able to remove existing mammospheres when added on Day 4. This could be attributed either to the inability of the compounds to penetrate the mammospheres or could indicate that they were only inhibitory during the early stages of mammosphere formation. This was supported by the fact that pre-treatment of MCF-7 cells with either RU017 or RU018 prior to seeding into anchorage-independent conditions had no effect on the ability of the cells to form mammospheres under these conditions. A possible explanation for this is that the effect of the compounds on the MCF-7 cells may be specific to their initial propagation under anchorage-independent serum-free growth conditions. This is supported by the fact that dissociated untreated mammospheres seeded into regular anchorage-dependent conditions and then treated with the algal compounds experience only a minor decrease in viability as opposed to the large decrease in viability seen when cells are treated with these compounds upon seeding into anchorage-independent conditions.

To further assess the efficacy of the algal compounds, RU017 and RU018, their effect on sphere forming efficiency was assessed for second and third generation mammospheres. In both cases, although the compounds were unable to prevent second or third generation mammosphere formation as was the case for primary mammospheres, they did result in a dose-dependent decrease in sphere forming efficiency. This reduced ability of the algal compounds to inhibit mammosphere formation in subsequent generations may be due to accumulated changes in growth characteristics of the cells as a consequence of prolonged culturing under anchorage-independent serum-free conditions, resulting in a more aggressive phenotype. This could also account for the increase in sphere forming efficiency in the vehicle control for the third generation as compared to that of the second generation. Interestingly, RU018 appeared more effective than RU017 in inhibiting second and third generation mammosphere formation, a finding which was consistent with the trend observed in terms of first generation mammosphere cell viability. This data implies that the stereochemistry of the monoterpene compounds plays a role in their mechanism of action against MCF-7 mammospheres.

The identification of CSCs within breast tumours has resulted in a major shift in focus in terms of the development of novel therapies to treat this disease [22]. It is now thought that complete eradication and prevention of relapse requires the removal of the stem cell subpopulation within a tumour [23]. Traditional treatments, such as chemotherapy and radiation, were originally developed to kill the rapidly dividing bulk population of cells within the tumour [22]. However, while these therapies are able to shrink the tumour, the effects are often transient and recurrence remains a reality for a substantial proportion of sufferers [22,24]. This has been attributed to the resistance of the CSC subpopulation to traditional therapies, such that these highly tumourigenic cells remain behind after chemotherapy or radiation treatment [23,24]. Therefore, there is a need to find agents which are able to specifically inhibit CSCs.

## Conclusions

The body of work described herein reports the first screening of marine algal compounds in a mammosphere assay. In particular, screening of the compounds FXN, RU017 and RU018 revealed that the latter monoterpene stereoisomers inhibited MCF-7 mammosphere formation. In contrast, the commonly used chemotherapeutic drug Ptx appeared to enhance both the formation and maturation of early mammospheres. More work is required to determine the specific molecular mechanisms mediating the mammosphere inhibitory activity of the halogenated monoterpenes as well as their respective cellular targets. In addition, the concentration of RU017 and RU018 required for such activity falls outside the druggable range and rational chemical modification is needed to improve their efficacy. Since the compounds form part of a structural series isolated from *Plocamium corallorhiza* and *Plocamium cornutum*[17], they could find application as tool compounds to study CSC selectivity in halogenated monoterpenes.

## Methods

### Reagents

Dulbecco’s Modified Eagle Medium (DMEM) containing Glutamax^™^, Ham’s F-10 Medium containing Glutamax^™^, foetal calf serum (FCS), B-27 supplement and penicillin-streptomycin-amphotericin (PSA) were obtained from Gibco (Invitrogen). Epidermal growth factor (EGF), basic fibroblast growth factor (bFGF), hydrocortisone, heparin, Accutase^®^, paclitaxel and fucoxanthin were from Sigma-Aldrich. Insulin was obtained from NovoRapid (Novo Nordisk Pharmaceuticals). The MTT and WST-1 Cell Proliferation kits were from Roche. Hoescht 33342 dye for flow cytometric analyses was obtained from Invitrogen, while the allophycocyanin (APC)-conjugated mouse anti-human CD24 and fluorescein isothiocyanate (FITC)-conjugated rat anti-human CD44 antibodies were from e-Biosciences, as were the isotype controls. The mouse anti-human Oct3/4 antibody was obtained from Santa Cruz, while the goat anti-mouse and goat anti-rabbit antibodies were from Sigma. The rabbit anti-histone H3 antibody was from Cell Signalling Technologies.

### Cell lines and culture conditions

The metastatic breast cancer cell line, MDA-MB-231 (ATCC: HTB-26) was maintained in culture in Dulbecco’s Modified Eagle Medium (DMEM) containing Glutamax^™^ and supplemented with 5% (v/v) heat-inactivated FCS, 100 U/ml penicillin, 100 μg/ml streptomycin and amphotericin (PSA) at 37&z.ousco;C in a humidified 9% CO_2_ incubator. The immortalized, non-transformed breast epithelial MCF-12A cells (ATCC: CRL-10782) were maintained using a 1:1 ratio of Ham’s F10 and DMEM containing Glutamax^™^ and supplemented with 10% (v/v) heat-inactivated FCS, PSA (as for MDA-MB-231 cells), 20 ng/ml EGF, 500 ng/ml hydrocortisone and 10 μg/ml insulin. The breast cancer cell line MCF-7 (ATCC: HTB-22) was maintained in culture either in anchorage-dependent or anchorage-independent conditions. In the former, cells were cultured as for the MDA-MB-231 line in regular 96-well plates.

### Mammosphere assay

Anchorage-independent growth was assessed by mammosphere assay, modified from that previously described [11]. Briefly, cells were lifted with 0.25% (v/v) trypsin in 0.61% (w/v) ethylenediaminetetracetic acid (EDTA), washed with phosphate-buffered saline (PBS) (137 mM NaCl, 2.7 mM KCl, 10 mM Na_2_HPO_4_, 2 mM KH_2_PO_4_ pH 7.4) and passed through a 40 μM cell strainer (BD Biosciences) to achieve a single cell suspension. Cell were seeded at a density of 1000 cells per well in ultralow attachment 96-well plates containing DMEM with Glutamax^™^ supplemented with 1% (v/v) PSA, 2% (v/v) B-27 supplement, 20 ng/ml EGF and bFGF, 4 ng/ml heparin and 10 μg/ml insulin. Treatment with either 0.61% (v/v) dimethyl sulphoxide (DMSO) vehicle control or paclitaxel (Sigma-Aldrich), fucoxanthin (Sigma-Aldrich), RU017 or RU018 was carried out either upon seeding or at Day 4 for quintuplicate samples. The cells were fed every 48 hours by the addition of fresh medium to existing culture volume and the resultant mammospheres photographed in triplicate using a Nikon camera (Coolpix 990) attached to a light microscope at 100 × magnification. Images were analyzed and scale bars calculated using ImageJ (NIH). Quantification in terms of Sphere Forming Efficiency (SFE) was carried out by counting of mammospheres under a light microscope at 10× magnification and reported as the number of mammospheres/spheres (average diameter = 100 μm) formed in 96 wells divided by the original number of single cells seeded and expressed as a percentage. Statistical significance for the various treatments was assessed using a Student’s *t*-test in GraphPad Prism (GraphPad Inc.).

Dissociation of mammospheres for cell viability assays and screening of second and third generation mammospheres was achieved using Accutase® solution (Sigma). Briefly, the mammospheres were collected by centrifugation, resuspended in 200 uL of 1 × Accutase® solution and incubated for 15 minutes before passing through a 40 μM cell strainer (BD Biosciences) to achieve a single cell suspension. Mammosphere-derived cells were seeded at a density of 6000 cells per well under regular anchorage-dependent conditions for cell viability analysis by MTT assay and at 1000 cells per well under anchorage-independent mammosphere culture conditions for second and third generation screening assays.

### Cell surface marker analysis by flow cytometry

Adherent MCF-7 cells were lifted using 0.25% (v/v) trypsin and washed with PBS, while anchorage-independent cells were collected by centrifugation (800 × *g*, 2 minutes), washed with PBS and dissociated using 0.25% (v/v) trypsin. In both cases, cells were resuspended to a final concentration of 5 × 10^6^ cells/ml in PBS and 5 × 10^5^ cells (100 μl) incubated with 0.5 – 1 μg of fluorophore-conjugated anti-CD44 and anti-CD24 antibodies or isotype matched control antibodies at 4 °C for 60 minutes. Unstained samples containing no antibodies were also included. The cells were washed twice with ice-cold PBS and collected by centrifugation as previously described, before analysis using a FACS Aria III flow cytometer. The fluorescein isothiocyanate (FITC) was excited at 488 nm and emission recorded in the 530/30 filter channel, while the 633 nm laser was used to excite the APC (allophycocyanin) fluorophore with emission recorded in the 660/20 filter channel (APC). Compensation values for FITC and APC fluorescence overlap were established using compensation controls and copied to all subsequent analyses. A total of 30 000 events were recorded for each sample. Flow cytometry data was analysed using FlowJo software (Tree Star Inc.). Isotype controls were used to establish fluorescence threshold gates with the gates set on the isotype control to exclude the major population of cells. Gates were then copied onto the respective samples in order to determine the CD44/CD24 expression profile of the sample. Two-colour dot plots were then constructed showing FITC (CD24) fluorescence on the y-axis and APC (CD44) fluorescence on the x-axis.

### Confocal microscopy analysis of anchorage-independent MCF-7 breast cancer cells

Anchorage-independent cells were seeded into 8-well chambered coverslips overnight. Cells were fixed by flash incubation in ice-cold methanol and left to air-dry before incubation in a blocking solution [1% (w/v) bovine serum albumin (BSA) in Tris-buffered saline (TBS)] at room temperature for 30 minutes. A primary antibody solution containing anti-CD44 conjugated to APC in 0.1% (w/v) BSA/TBS was added to the cells and incubated overnight at 4&z.ousco;C. Cells were washed twice with agitation in blocking solution, after which the cells were washed in distilled water containing 1 μg/ml of DNA binding dye Hoechst 33342. The coverslips were mounted onto glass slides using Dako mounting media (Dako). Differential contrast (DIC) and fluorescent images were taken using a Zeiss LSM510 meta confocal microscope.

### Western blot analysis

MCF-7 cells were seeded at a density of 4000 cells per well in anchorage independent mammosphere conditions and mammospheres allowed to develop over 8 days. In addition MCF-7 cells were cultured in anchorage-dependent regular culture conditions and lifted using 0.25% (v/v) trypsin. Whole cell lysates were prepared from adherent and mammosphere cultures. Briefly, cells were collected by centrifugation at 2000 rpm for 3 minutes and resuspended in 100 μM SDS sample buffer (125 mM Tris–HCl pH 6.8, 2% (w/v) SDS, 20% (v/v) glycerol, 0.2% (w/v) bromophenol blue) before boiling for 5 minutes. Thereafter, 30 ug of protein from each lysate was loaded onto a 12% SDS polyacrylamide gels, separated by electrophoresis and transferred onto a nitrocellulose membrane, before probing with mouse anti-Oct3/4 (1 in 2000) at 4&z.ousco;C overnight. The HRP-conjugated anti-mouse secondary antibody was detected using the ECL Advanced Western Blotting Kit (GE Healthcare) and visualized using the Molecular Imager ChemiDoc XRS System (BioRad). Lysates were probed with rabbit anti-histone H3 antibody (1 in 2500) as a loading control.

### Extraction of compounds from marine algae

Specimens of the red alga *Plocamium cornutum* were gathered off the south-east coast of South Africa and the halogenated monoterpenes extracted as previously described [25].

### Cell viability assays

Cell viability after treatment with the compounds was evaluated by means of the MTT assay in the case of adherent cells and WST-1 assay in the case of mammospheres, according to manufacturer’s instructions. For both assays, percentage viability after treatment with compounds was calculated relative to the DMSO vehicle control (taken as 100% viability) when equal numbers of cells are seeded. For the MTT Cell Proliferation Kit 1, MDA-MB-231, MCF-7 or MCF-12A cells were seeded at a density of 6000 cells/well in 96-well plates and assessed for viability as previously described [17]. In the case of the WST-1 kit, after treatment and growth of the mammospheres for 8 days, 10 μL of a 5 mg/ml WST-1 reagent was added to each well and incubated for a further 4 hours before reading the absorbance at 450 nm. Viability assays were carried out in quintuplicate and statistical significance assessed using GraphPad Prism.

## Abbreviations

APC: Allophycocyanin; BCSC: Breast cancer stem cell; BSA: Bovine serum albumin; CSC: Cancer stem cell; DMEM: Dulbecco’s Modified Eagle Medium; DMSO: Dimethyl sulphoxide; EGF: Epidermal growth factor; FCS: Fetal calf serum; FGF: Fibroblast growth factor; FITC: Fluorescein isothiocyanate; FXN: Fucoxanthin; PBS: Phosphate-buffered saline; PSA: Penicillin-streptomycin-amphotericin; Ptx: Paclitaxel; SFE: Sphere forming efficiency; TBS: Tris-buffered saline

## Competing interests

The authors declare that they have no competing interests.

## Authors’ contributions

ALE, JdlM and GLB were involved in the conception and design of the study. JdlM and ALE drafted the manuscript. JdlM carried out the MCF-7 mammosphere assays and screening of marine compounds. JNS carried out the flow cytometry and MGS the Oct4 Western analysis. MTC and DRB were responsible for the purification of the compounds from marine algae. All of the authors read and approved the manuscript.
